# Marrying Story with Science: The Impact of Outdated and Inconsistent Breast Cancer Screening Practices in Canada

**DOI:** 10.3390/curroncol29050286

**Published:** 2022-05-13

**Authors:** Jennie Dale, Michelle Di Tomaso, Victoria Gay

**Affiliations:** 1 Independent Researcher, Toronto, ON, Canada; 2 Independent Researcher, Vancouver, BC, Canada; michelleditomaso70@gmail.com (M.D.T.); victoria@victoriagayconsulting.com (V.G.)

**Keywords:** breast, cancer, screening, dense, patient advocacy, breast density, breast density notification, Canada

## Abstract

Behind the science of breast cancer in Canada, as well as globally, are the stories of thousands of women, their families, and their communities. These include stories from those who have died or those suffering from the realities of stage III and stage IV breast cancer due to late detection, misinformation, and dismissal. The reality for these women is that, whilst grateful for the latest developments in cancer research, much of this knowledge is not reflected in policy and practice. Canadian guidelines do not reflect the recommended screening by experts within the field and inequities in screening practices and practitioner knowledge exist in different areas within Canada. Told through the stories of women with lived experiences of late-stage breast cancer and supported by scientific evidence, this paper explores the impact of outdated breast cancer screening practices on the lives of women. Recent patient advocacy is driving changes, such as notifying women of their breast density in a few jurisdictions in Canada, but we call for the whole medical community to take responsibility and ensure breast screening is optimised to save more lives.

## 1. Introduction

**Story 1.** *In 2019, a 46-year-old finds a lump in her breast and speaks to her family doctor. She is referred for a mammogram and ultrasound, then booked in for a biopsy of an 8 cm lump; within a few weeks, she is diagnosed with Triple Positive de novo stage IV breast cancer. This is after being denied regular mammograms by her family doctor at the age of 40 because the screening program in the province where she resides, Alberta, requires a requisition for the first screen of patients in their 40s and only begins self-referralat the age of 50. This is also after being repeatedly monitored using mammography for one existing lump when she lived in British Columbia in her 30s, and after practitioners had paperwork showing that her Volpara Breast Density Score is D, meaning a decreased sensitivity of mammography to identify cancerous masses and an increased risk of breast cancer. If this woman had not moved from the province of British Columbia to Alberta, she could have self-referred for screening from the age of 40 and possibly learned she had cancer before the Triple Positive breast cancer had spread to her lymph nodes, spine, sternum, and ribs. She would also be aware of her breast density category and the risks associated with this, as they are reported on the patient-facing screening documentation. Therefore, it is unlikely she would have been given a 22% prognosis of living for the next five years. That was three years ago. She has 2 children, aged 5 and 8.*

Regrettably, this example of a lack of information on breast density, inconsistencies in screening practices between geographical jurisdictions (ten provinces and three territories) in Canada, and dismissal by the healthcare system is not unique. In North America, it is estimated that 5% of women with breast cancer will be diagnosed with de novo stage IV (metastatic) disease [1]. Statistics from the United States suggest that, of new diagnoses of metastatic breast cancer, 26% are de novo (12,966 in the US in 2013) [2]. Late-stage breast cancer (stages III and IV) results in increased morbidity, more intensive chemical and surgical treatment, and increased mortality (Table 1) [3,4,5,6]. With optimal screening practices and knowledge-sharing, combined with improved education and awareness between practitioners and patient communities, the number of late-stage diagnoses can be reduced. 

Dense Breasts Canada is a national education and advocacy organisation committed to raising awareness about the risks associated with dense breasts, advocating for breast density notification and optimal breast cancer screening. Over the past six years, Dense Breasts Canada has collated the stories of women in Canada who were not informed of their breast density, denied mammograms, and dismissed by healthcare practitioners. These stories of screening, diagnosis, and treatment experiences represent an important component of the breast cancer landscape that we believe should be central to research, knowledge translation, guidelines, policy, and practice. 

Concerningly, the stories shared with Dense Breasts Canada highlight five key shortcomings in the screening and diagnosis of breast cancer in Canada: (i) the impact of outdated screening guidelines from the Canadian Task Force on Preventive Health Care (Canadian Task Force); (ii) inconsistencies between the Canadian Task Force breast cancer screening guidelines and those of the jurisdictions across Canada, creating confusion for medical practitioners and patients; (iii) inequities in screening practices between jurisdictions in Canada, resulting in an increased risk for women in a few provinces; (iv) limited awareness among practitioners and the general public of breast density risks and screening options; and (v) the dismissal of women by medical practitioners. 

This paper looks at these five issues through stories of women with breast cancer to bring a personal lens to the science and policy, and to highlight the pressing need for the medical community to collectively advocate for the current science to match the policy, knowledge, and practice. 

## 2. Marrying Stories and Science

### 2.1. The Impact of Outdated and Inconsistent Screening Guidelines

**Story 2.** *In 2020, a woman in Ontario feels a thickening of her breast tissue. She is 47. She thinks it is likely due to premenopausal changes. It develops into a dimple, so she visits her family doctor for an exam. He refers her for a mammogram. She is called back the next day for another mammogram and a biopsy. She has no history of breast cancer in her family and is shocked at the callback. The lumpectomy and sentinel node biopsy reveal stage I grade 3 breast cancer. Radiation treatment and tamoxifen are planned. At her preradiation CT scan, two additional tumours are found in her lungs and pancreas. She has an extensive Whipple procedure (pancreaticoduodenectomy) to remove the tumour in her pancreas, which also removes part of her small intestine, gall bladder, and pancreatic duct. The pathology reveals that the pancreas and lung tumours have metastasized from the breast cancer. Her diagnosis is updated to de novo stage IV breast cancer. It takes six months to recover from her surgery. She stops her career as an intensive care nurse and goes on long-term disability. She also has surgery to remove her ovaries, which pushes her into the menopause. She takes the drugs Letrozole and Ibrance, which cause fatigue and mouth sores. She experiences ongoing digestive issues. She loses contact with many friends. Check-ups show that the cancer has stabilised. The median survival rate for women with metastatic breast cancer is three years. Her diagnosis was two years ago.*

The Canadian Task Force publishes guidelines for breast cancer screening [7]. The screening guideline for women aged 40–49 is significantly based on the Canadian National Breast Screening Study (CNBSS) [8,9], which concluded that mammography for women in their 40s did not reduce breast cancer deaths. This contributed to the recommendation that advised against mammograms for women in their 40s [7,10]. These guidelines were adopted by the Ontario Breast Screening Program, which is why this 47-year-old patient was not screened in her 40s.

Since the initial publication of the CNBSS, there has been a suite of evidence questioning the study protocol validity, particularly the likelihood of compromised randomisation [11,12,13,14,15,16,17]. Since its publication, the evidence has also shown the benefits of improved screening regimes. For example, Arleo and Hendrick [18] used modelling to demonstrate that the most lives are saved by annual screening starting at the age of 40; Coldman and Phillips [19] showed that women who screened between 40–49 years of age were 44% less likely to die of breast cancer than women who did not; Oeffinger [20] estimated that 27% of the total years of life lost to breast cancer were a result of cancers that are detectable between the ages of 40 and 49; and Webb and Cady [21] found that the median age at diagnosis of fatal cancers was 49 years and most deaths from breast cancer occurred in unscreened women. Even greater mortality risks are experienced by Black, Asian, Native American, and Hispanic women, with a younger age of onset (mid-to-late forties in comparison with mid-sixties for Caucasian women) and a higher incidence of aggressive breast cancer [22,23,24]. These studies suggest that, to maximise mortality reduction and life-years gained, regular screening needs to start before the age of 50.

As the Canadian Task Force guidelines consider only the results from randomised clinical trials [7] and it would not be ethical to assign women to a non-mammography control arm, these guidelines will remain unchanged until we successfully advocate for these guidelines to change or for the CNBSS to be retracted.

The Canadian Task Force also only considers mortality reduction as a benefit of screening. Looking beyond mortality, regular mammography screening from the age of 40 is associated with a decreased stage at diagnosis and the receipt of less extensive treatments as well as a reduced need for chemotherapy, reduced mastectomies, and increased breast-conserving surgery [3,4,5,25,26]. Considering this scientific evidence, and the significant surgical, chemical, and hormonal treatment the woman in this story had to endure due to the late diagnosis, it is essential that quality of life, treatment options, and surgery are also considered in screening recommendations [5]. 

The Canadian Society of Breast Imaging recommendations for optimal screening to reduce mortality and morbidity [27] (Table 2) vary considerably from the Canadian Task Force Recommendations. As these are the recommendations of the experts within the breast radiology community and are based on current scientific evidence, we recommend they be adopted.

### 2.2. Differences between Provincial and Canadian Task Force Breast Cancer Screening Guidelines Create Confusion for Medical Practitioners and Patients

**Story 3.***A 50-year-old woman originally from Brazil moves to Canada at the age of 40. She is aware of her breast density, has a history of finding benign cysts in her breasts, and has had regular mammograms, starting at the age of 35, until she moves to Canada. On multiple occasions, she speaks to her family doctor in British Columbia about screening mammography, but is repeatedly discouraged and quoted the Canadian Task Force screening guidelines that recommend mammograms every 2 years from the age of 50, rather than the provincial guidelines in British Columbia, which allow for self-referral at the age of 40. She has had progressively worsening hip pain from the age of 47 and repeatedly visits the family doctor for this reason; she is referred to a physiotherapist and told to practice yoga. Upon a worsening of the symptoms and seeing a different physician, she has an X-ray then CT scans and biopsies, which reveal stage IV breast cancer with two nodules in her right breast, multiple lesions in the pelvic bones and greater destruction to the hip socket, iliac, and ischium bones as well as multiple nodules in her lungs*.

Although the Canadian Task Force breast screening guidelines currently do not recommend screening until the age of 50, screening varies considerably between the ten provinces and three territories of Canada. For example, in the province of British Columbia, women can self-refer every 2 years from the age of 40 [28], yet, as women do not receive invitation letters to the screening program, most are not aware of this opportunity. A survey of 2530 women in Canada showed that 42% of respondents were unaware of the age they were eligible for screening [29]. Conflicting messages between the Canadian Task Force and provincial guidelines, as well as the information available to practitioners and patients may have led in the above case to the diagnosis of de novo metastatic breast cancer. 

The Canadian Task Force emphasises the potential harms caused by “false-positives” (when women are recalled for further examination because of radiological signs on the screening examination that then turn out to be normal or benign), suggesting that screening leads to physical and psychological consequences that are a greater risk for women under 50 years of age [7]. A review of surveys concluded that the level of short-term stress that being recalled caused did not reach that of clinical anxiety [30], and many studies have shown no evidence of long-term distress in recalled women with “false-positive” mammograms [31,32,33]. A survey of women by the Canadian Task Force confirmed that reductions in breast cancer mortality outweighed any recalls or overdiagnosis [34], but this was dismissed by the authors, who suggested that the women surveyed did not have enough information to make this conclusion [35]. An emphasis on the harm of mammograms due to unnecessary anxiety ignores the reassurance received by the majority of patients and the scientific literature that underlines the benefits of regular screening. Yet, this is not widely translated to medical practitioners across Canada. This is also demonstrated in Story 3 and the 47-year-old diagnosed with stage IV breast cancer as well as in hundreds of stories from women across Canada collected in the recent survey by Dense Breasts Canada [29]. 

“Even with women being able to self-refer for mammograms between ages 40 and 50 in BC, I think that there needs to be more education, including and perhaps most importantly, of family doctors regarding symptoms, the need for an earlier baseline mammogram, breast density effects on not only reducing how effective mammograms can be at detecting tumours but also predisposing women to breast cancer, so that misguided information will not end up leading to late diagnoses and deaths that could have been prevented.” 50-year-old patient diagnosed with stage IV breast cancer at the age of 47.

### 2.3. Geographical Inequities in Screening across Jurisdictions Mean Several Women Risk a Late Diagnosis Based on Where They Live

**Story 4.** *A 50-year-old mother of three in Ontario finds a lump in her left breast before she is scheduled for her first mammogram, which is available for women in Ontario from the age of 50. She has previously inquired about mammograms in her 40s due to a family history of breast cancer (maternal and paternal aunts, and a first cousin who was diagnosed premenopausal), but has been informed that she “did not qualify under the rules and, by implication, should not worry”. After discovering the lump, she has a mammogram, which detects a vague architectural distortion. She has an ultrasound that shows three masses and an MRI, which reveals five. The post-surgical pathology reveals that there are actually nine tumours in one breast. Cancer is also found in most of her lymph nodes on the same side. She is diagnosed with stage III breast cancer. She is never informed of her Category D density. She has a radical mastectomy of the left breast, a full nodal dissection on the left side, and chemotherapy. In addition to the scar tissue, irreversible tightness in the chest muscles, hair loss, nausea, and fatigue associated with chemotherapy, her treatment pushes her into the menopause, accelerating the effects of aging, reducing her peak cardio fitness, and increasing her risk of osteoporosis. She is required to take an aromatase inhibitor daily.*

Differences in provincial screening protocols across Canada result in women such as this 50-year-old being diagnosed with later stage breast cancer because of where she lived. Jurisdictional variations in screening for women between the ages of 40 and 49 vary dramatically [36] (Table 3). For example, women aged 40 who live in Nova Scotia can self-refer to the screening program for annual screening. If they lived in other provinces, such as Saskatchewan or Quebec, they would need a diagnostic requisition from their healthcare practitioner. 

Had this woman lived in British Columbia, Nova Scotia, Yukon, or Prince Edward Island—where women can self-refer at the age of 40 and, for the latter three, annually (Table 3)—her cancer may have been found earlier. 

Jurisdictional variations in screening relate not only to the age at which women are eligible for screening (40 or 50), but the age at which screening stops (69 in Quebec; 74 in other jurisdictions) and the frequency of screening (annual or biennial) as well as variations due to different risk factors, such as breast density.

Furthermore, there are no national guidelines for screening individuals at a “high risk” and screening protocols vary across jurisdictions [37]. The definition of a high risk of developing breast cancer also varies across Canada. It is up to the individual province whether women are considered to be high risk based on a lifetime risk of 20–25%. 

Although the Canada Health Act suggests that people in Canada should have “uniform access to insured health services, free from financial or other barriers” [38], this is not the case; access to breast cancer screening—and, therefore, the risk of a late-stage diagnosis—varies depending on where women live in Canada.

### 2.4. Limited Awareness of Breast Density Risks and Screening Options

**Story 5.** *A 36-year-old woman finds a lump on her right breast. After a mammogram, it is deemed to be benign and disappears over time. She is informed that she has dense breasts, but given no information about what this might mean. She assumes it is positive and related to her healthy and fit physique. Eighteen years later, at fifty-four, she finds another lump in the same breast. She had a clear mammogram six months earlier as part of the biennial screening program in Ontario, but has not been informed of her breast density or any associated risks through either the screening mammogram results letter she received or at any screening appointments or follow-ups. Upon the examination of her lump, her family doctor refers her for a mammogram and ultrasound, followed by a biopsy. She has a lumpectomy and sentinel node biopsy, revealing Triple Negative stage III aggressive grade 3 breast cancer. Her diagnosing physician tells her that the cancer has probably been developing for quite a time, but was likely missed on the previous mammogram because of her heterogeneously dense breasts. She receives chemotherapy, the removal of 17 more lymph nodes, a prophylactic bilateral mastectomy, and 25 rounds of radiation. An aggressive treatment plan is designed to target the late-stage breast cancer, which may have been detected sooner if breast density had been considered and supplemental screening performed.*

Approximately 43% of women over the age of 40 have dense breasts [39]. Dense breasts are a risk factor of greater prevalence than family history [40] and pose two risks: an increased risk of breast cancer and an increased risk that the cancer will be masked on a mammogram by dense tissues [41]. Women with dense breasts are significantly more likely to be diagnosed with an interval cancer [42]. In Canada, only six jurisdictions provide information on breast density directly to all women having a screening mammogram (Table 4). In five jurisdictions, only women in Category D of density are informed, even though both Category C and D are considered to be dense and associated with an increased risk of breast cancer and difficulty in detection through mammograms. This means that a large percentage of women in Canada are still not being informed of their breast density and are denied the opportunity to be proactive about their breast health.

Although it is encouraging that women in a few jurisdictions are now being told their breast density in the results letters mailed by the provincial screening programs, not all correspondence provides information on the associated risks of dense breasts. In addition, only six provinces offer annual mammograms for women with category D density. Seely and Peddle [43] compared interval cancer rates in provinces where mammography is performed biennially to provinces that recall women with the highest density annually. They showed that provinces screening women with dense breasts annually had fewer interval cancers.

In addition, only women in British Columbia and Alberta have relatively accessible supplemental screening. Evidence since 1995 has shown that an ultrasound finds additional cancers missed by mammograms [44,45,46] and reduces the interval rates and rates of late-stage disease [47,48]. Wu and Warren [49] found 7 additional cancers per 1000 women via a screening ultrasound in women with dense breasts. Of those, 40% of the cancers were in women with no family history and 60% were in women with category C density.

Had the above-mentioned woman lived in British Columbia or Alberta, where women with dense breasts are informed of their breast density and can access a screening ultrasound more easily, her cancer may also have been found much earlier. The differences in breast density notifications in the jurisdictions across Canada, combined with the gaps between the scientific evidence and practices, are likely impacting on the lives of women.

### 2.5. Dismissal of Women

**Story 6.** *A 42-year-old woman in Alberta starts experiencing back pain whilst walking. It does not improve. She has an X-ray, which comes back clear, and is referred to a physiotherapist and chiropractor. The pain persists and worsens over the next year to the extent that she has to stop work. During physiotherapy exercises, she hears a popping noise and experiences excruciating pain. She is referred for another X-ray, which shows arthritic changes, but with no explanation as to the cause. She does not think it could be breast cancer as she was dismissed by her family doctor as “too young” when she requested a mammogram. Serendipitously, she reads an article about a woman with metastatic breast cancer with no obvious symptoms apart from back pain. She does a self-exam and finds a lump. In quick succession, she has a mammogram, ultrasound, biopsy, and MRI (privately paid). She learns that the breast cancer has metastasized throughout her bones, liver, and lymph nodes. She is diagnosed with stage IV Invasive Ductal Carcinoma, hormone negative, and Her2-positive cancer. She has surgery to insert rods in both of her femurs, spends six weeks in the hospital, and has radiation targeting her pelvis and femurs as well as six rounds of chemotherapy. She has ongoing targeted therapy every three weeks. She uses a wheelchair and a walker. She is 46. She has three teenage children.*

Although not explored widely in the literature, the dismissal of women with breast cancer symptoms can be inferred in the number of malpractice cases for delays in breast cancer diagnoses. It represents a major number of malpractice claims in the UK [50]. In the USA, it is the second most common cause of legal medical malpractice suits and the largest total indemnity pay-out by medical insurance companies; two-thirds of these claims involve women aged 50 and younger [51,52,53,54].

Murphy et al. [55] reviewed 264 cases of litigation about breast cancer care, of which 59% related to delays in diagnosis. Allen and Petrisek [51] investigated the evidence of dismissal by healthcare practitioners upon common signs of breast cancer, such as the identification of lumps and nipple discharge. Several women in this study indicated that “physicians failed to recognize symptoms, neglected to perform diagnostic procedures and provided erroneous information because they were unwilling to believe that younger women were likely to experience this illness” [51]. According to a systematic review, this is a common reason for a delayed diagnosis in premenopausal women [56]. Stories such as these are also explored by Dense Breasts Canada [29] and mirror the case study provided in this section. 

Delays in diagnoses are not always attributable to practitioners. The reasons for women delaying the seeking of a diagnosis after the identification of symptoms have also been explored [56,57,58,59] and are estimated to represent a minority of cases of 20–30% [60]. However, a previous dismissal, lack of respect, and symptoms not taken seriously by healthcare practitioners for breast-related or other health issues in the past have been contributing factors in many cases [58]. A delayed diagnosis due to repeated practitioner dismissals has been demonstrated for other health issues of women, including endometriosis, with an average of 7–10 years to diagnosis [61,62], and premenstrual dysphoric disorder, with an average of 20 years [63], as well as a spectrum of other medical conditions explored by Dusenbery [64]. Such dismissal is commonly attributed to the perception of medical issues of women being influenced by emotional factors [65,66]. 

Although the persistent dismissal of the health issues of women is a much larger issue, it is evident that there is a considerable need to address delays in breast cancer diagnoses related to gaps in the current knowledge and communication between healthcare practitioners and patients.

## 3. Discussion

The stories and issues presented in this paper are emblematic of the flaws in the screening policies in Canada. The 46-year-old diagnosed in Story 1 with de novo stage IV breast cancer who was denied a mammogram in her 40s due to the specific screening practices within her province was dismissed on multiple occasions by her family doctor and was not informed of her breast density. Women in their 40s are not acceptable losses, particularly considering that 17% of breast cancer cases occur in this age group [67]. Many of these women have young children, are caring for aging parents, and are contributing to the economy.

Dense Breasts Canada has successfully advocated for patient notification of breast density, and ten jurisdictions (Table 4) have made changes to their practice over the past six years, but, as the stories demonstrate, more must be done to ensure access to the early detection of breast cancer for all women in Canada. We are asking for support from the medical community in advocating for: (a) updated guidelines for breast cancer screening; (b) sharing current evidence with all healthcare providers; and (c) tracking the incidence of metastatic breast cancer.

Updated policies in Canada based on scientific evidence of reduced mortality and morbidity would include: self-referral for annual mammograms across jurisdictions starting at the age of 40; directly informing all women having a screening mammogram of their breast density and the associated risks of dense breasts; and offering annual supplemental ultrasound screening (in addition to mammograms) to all women with dense breasts (Category C and D), regardless of family history. Additionally, high-risk women should be identified and offered supplemental MRI where available. 

Furthermore, although this study focuses on Canada, we recommend that internationally updated guidelines be based on the latest scientific evidence. The documentation suggests that breast screening policies in other countries have been influenced by the outcomes of the CNBSS, which is cited in screening guidelines and recommendations for the US [10], UK [68], Europe [69,70], and Australia [71]. The CNBSS study has been discredited [12] and should be retracted from the medical literature. Oncologists and other breast cancer specialists globally can advocate for updated screening guidelines. 

We recognise that advocacy will not change outdated screening guidelines and practices overnight. To reduce the incidence of stage IV breast cancer, medical professionals, advocates, and patients need to be presented with the benefits and limitations of screening based on current evidence. This specifically includes: directly addressing gaps in education relating to breast density; the benefits of screening at the age of 40 and breast cancer in younger women; and an increased awareness of eligibility of women for screening. Advocacy is the responsibility of all stakeholders in the breast cancer community. Contradictions in the guidelines compared with the latest evidence can be presented at local, national, and international conferences of medical practitioners; specifically, nurses and family practitioners. Information can be included in the content for continuing medical education sessions. Importantly, whilst women are referred to oncologists at the stage of breast cancer identification and treatment, conversations with patients are essential to highlight the latest evidence of screening. 

Finally, we need to track the incidence of stage IV breast cancer across the jurisdictions in Canada to actively support the development of policies and practices that target a reduction in the incidence of late detection. This has recently been implemented in the province of Quebec where the incidence of stage IV breast cancer is being tracked to support research into screening, diagnosis, manifestation, and treatment [72]. 

Our article includes six stories from women with stage III and de novo stage IV breast cancers to demonstrate the significant impacts of misguided policies as well as misinformation among the medical community and patients. These stories were chosen as they exhibit the impacts of late diagnosis, but there are also many positive stories of screening and early detection.

**Story 7.** *A 41-year-old woman living in Prince Edward Island self-refers for a mammogram, which leads to the detection of stage I Invasive Ductal Carcinoma. It is confirmed by the general practitioner and surgeon that the tumour could not have been identified by a physical examination. She has surgery to remove the tumour and sample the lymph nodes, 21 rounds of radiation, and hormone therapy scheduled for the next 5–10 years. She knows that access to self-referral for screening from the age of 40 in her province allowed her breast cancer to be found early.*

**Story 8.** *A 40-year-old woman from British Columbia is encouraged by her family doctor to have a screening mammogram to obtain a “baseline” and understanding of her breast density. Her breasts are identified as dense, and an abnormality is detected. She has a diagnostic mammogram, ultrasound, and then biopsy and is diagnosed with stage I breast cancer 17 days after her initial screening mammogram. She has a lumpectomy, completes 20 rounds of radiation, and receives hormone therapy, which is scheduled for the following 5 years. She is grateful to have been able to self-refer for screening at the age of 40, and that her proactive family doctor recommended her to go.*

## 4. Conclusions

Using a series of case studies, we have highlighted the impacts of suboptimal cancer screening on the lives of women. We highlighted stories of late diagnosis due to: outdated and inconsistent screening guidelines from the Canadian Task Force; inconsistencies between those guidelines and those of individual jurisdictions, creating confusion for medical practitioners and patients; geographical inequities in screening between jurisdictions, resulting in an increased risk for women in a few provinces; limited awareness of both medical professionals and patients of the risk of dense breast; and the dismissal of women by medical practitioners. 

We ask for the medical community to advocate for better policies by: (a) individually and collectively asking for breast screening guidelines to be updated to reflect the latest scientific evidence; (b) information-sharing between the medical community and patients; and (c) the national and provincial collection of stage IV breast cancer incidences. 

Action from our whole community to advocate for optimal screening practices will help to reduce mortality from breast cancer as well as reduce the incidence of stage III and de novo stage IV breast cancers, reduce aggressive treatment and surgery, lessen the need for chemotherapy, and increase the quality of life for women with breast cancer. 

## Figures and Tables

**Table 1:** The relative 5-year survival of breast cancer patients by stage [6].

Stage	Relative Survival (%)
Stage I	100
Stage II	93
Stage III	72
Stage IV	22

**Table 2:** Comparison of breast screening recommendations from The Canadian Task Force on Preventive Health Care [7] and the Canadian Society of Breast Imaging recommendations [27].

Breast Screening Recommendations: Canadian Task Force on Preventive Health Care	Recommendations: Canadian Society of Breast Imaging and Canadian Association of Radiologists
Screening for women aged 40–49 is not recommended	Women aged 40–49 should screen annually with mammography
Women aged 50–74 should screen every 2–3 years with mammography	Women aged 50–74 should screen every 1–2 years with mammography
There are no recommendations for screening women over age 74	Women over aged 74 should screen every 1–2 years with mammography as long as they are in good health with life expectancy of ~7+ years
Supplemental screening is not recommended for women with dense breasts	Women with dense breasts can benefit from a supplemental screening
Risk assessment is not recommended	Risk should be assessed by age 25–30 to determine if early screening is appropriate
Clinical breast exam is not recommended	Mammography may miss breast cancers and a clinical breast exam is complementary to mammography
Breast self-exam is not recommended	Breast self-awareness is recommended

**Table 3:** Screening differences between jurisdictions in Canada for women aged 40–49 [36].

Province/Territory	Can Self-Refer at Age of 40	Can Self-Refer Annually in their 40s	Need a Requisition from Ages 40–49
British Columbia	Yes		
Nova Scotia	Yes	Yes	
Prince Edward Island	Yes	Yes	
Yukon Territory	Yes	Yes	
Alberta			1st screen only
Manitoba			Yes
New Brunswick			Yes
Saskatchewan			Yes
Ontario			Yes
Newfoundland			Yes
Quebec			Yes
North West Territories			1st screen only
Nunavut (no program)			

**Table 4:** Breast density notification differences between jurisdictions in Canada [36].

Province/Territory	All Women Having a Screening Mammogram Are Mailed Their Breast Density in Results Letter	Only Women in Category D Are Told Their Density	Women in Category D Are Offered Annual Mammograms
British Columbia	Yes		
Nova Scotia	Yes		
Prince Edward Island	Yes		Yes
Yukon Territory		Yes	Yes
Alberta	Yes		
Manitoba	Yes		
New Brunswick	Yes		
Saskatchewan		Yes	Yes
Ontario		Yes	Yes
Newfoundland		Yes	Yes
Quebec			
North West Territories		Yes	Yes
Nunavut (No program)			

## Data Availability

Not applicable.
